# A Mobile Technology Intervention With Ultraviolet Radiation Dosimeters and Smartphone Apps for Skin Cancer Prevention in Young Adults: Randomized Controlled Trial

**DOI:** 10.2196/mhealth.9854

**Published:** 2018-11-28

**Authors:** Elke Hacker, Caitlin Horsham, Dimitrios Vagenas, Lee Jones, John Lowe, Monika Janda

**Affiliations:** 1 Institute of Health and Biomedical Innovation School of Public Health and Social Work Queensland University of Technology Brisbane Australia; 2 School of Health and Sport Sciences Faculty of Science, Health, Education and Engineering University of the Sunshine Coast Maroochydore Australia; 3 Centre of Health Services Research Faculty of Medicine The University of Queensland Brisbane Australia

**Keywords:** health promotion, melanoma, preventive medicine, public health, skin neoplasms, mobile phone, sunburn, sunlight, technologies, Web apps

## Abstract

**Background:**

Skin cancer is the most prevalent and most preventable cancer in Australia. Despite Australia’s long-running public health campaigns, young Australian adults continue to report high levels of ultraviolet radiation (UVR) exposure and frequent sunburns. Young people are now increasingly turning away from traditional media, such as newspapers and TV, favoring Web-based streaming, which is challenging the health care sector to develop new ways to reach this group with targeted, personalized health promotion messages. Advances in technology have enabled delivery of time- and context-relevant health interventions.

**Objective:**

The primary aim of this randomized controlled trial was to test the effect of UVR feedback from a smartphone app or a UVR dosimeter feedback device on sun protection habits, sun exposure behaviors, sunburn, and physical activity levels in young adults.

**Methods:**

Young adults aged 18-35 years (n=124) were recruited from Queensland, Australia, between September 2015 and April 2016, via social or traditional media campaigns and outreach activities in the local community. Participants were randomized into 3 groups for a 4-week intervention: (1) no intervention control group; (2) UVR monitor group, who were asked to wear a UVR dosimeter feedback device set to their skin type; and (3) a SunSmart app group, who were asked to download and use the SunSmart phone app. Data were self-assessed through Web-based surveys at baseline and 1 week and 3 months postintervention.

**Results:**

Complete data were available for 86.2% (107/124) of participants (control group, n=36; UVR monitor group, n=36; and SunSmart app group, n=35). Intervention uptake in the UVR monitor group was high, with 94% (34/36) of participants using the device all or some of the time when outdoors. All SunSmart app group participants downloaded the app on their smartphone. There was no significant difference in the change in the sun protection habits (SPH) index (main outcome measure) across the 3 groups. However, compared with the control group, a significantly greater proportion of the participants in the UVR monitor group reduced their time unprotected and exposed to UVR on weekends during the intervention compared with the baseline (odds ratio [OR]: 2.706, 95% CI 1.047-6.992, *P*=.04). This significant effect was sustained with greater reductions observed up to 3 months postintervention (OR: 3.130, 95% CI 1.196-8.190, *P*=.02). There were no significant differences between the groups in weekday sun exposure, sunscreen use, sunburn, suntan, or physical activity.

**Conclusions:**

Using technology such as apps and personal UVR monitoring devices may improve some sun exposure behaviors among young adults, but as the SPH index did not increase in this study, further research is required to achieve consistent uptake of sun protection in young people.

**Trial Registration:**

The Australian and New Zealand Clinical Trials register ACTRN12615001296527; https://www.anzctr.org.au/Trial/Registration/TrialReview.aspx?id=368458 (Archived by WebCite at http://www.webcitation.org/731somROx)

## Introduction

Ultraviolet radiation (UVR) or sunlight exposure is the main environmental risk factor for melanoma and keratinocyte skin cancers (including basal cell carcinoma and squamous cell carcinoma). It is predicted that in the United States, new cases of melanoma will rise from around 70,000 in 2007-2011 to 116,000 in 2026-2031 [1]. Melanoma is the most common cancer in those aged 15-39 years in Australia [2]. Consistently across the United States, several European countries, and Australia, young adults are reporting higher levels of sunburn compared with older adults, despite having good knowledge and sun-protective intentions [3-5]. In Australia, people aged 18-24 years were seven times more likely to report sunburn on the previous weekend than those aged >65 years [6]. Young people, men, and those from a lower socioeconomic class or education level are all less likely to engage in preventive activities [7].

Over the past 30 years, Australia has successfully implemented world-class skin cancer prevention campaigns such as Slip! Slop! Slap!, SunSmart, and “Protect yourself in five ways from skin cancer” delivered mostly using traditional public health and media channels such as posters, brochures, television, radio, and newspaper advertising [8,9]. These programs have raised public awareness and improved preventive behaviors among Australians and have thought to have led to a slight reduction in melanoma incidence in younger generations [10]. Despite this success, the achievable impact of traditional media is waning due to the increased use of personalized internet-delivered multimedia content, especially among young people [11-13].

While the increasing use of mobile technology offers many opportunities for providing and collecting information and delivering time-, person-, or context-sensitive health interventions, very few studies to date have tested sun protection interventions with personalized messaging [14]. Buller et al provided personalized time until sunburn information using a mobile phone app to >600 US residents, aged above 18 years, which led to a marked increase in sun protection behavious [15]. A greater proportion of intervention group participants reported they kept time in the sun to a minimum (60% for app users vs 49% for nonusers; *P*=.04) and used more sun protection (39% vs 34%; *P*=.04). Our previous study recruited 574 participants aged 18-42 years from Queensland, Australia, sending 21 personalized motivational sun protection short message service text messages [16]. At 12 months postrandomization, sun protection group participants (mean change, 0.12) had significantly greater improvement in their sun protection habits (SPH) index than the physical activity attention control group participants (mean change, 0.02; *P*=.03) [16]. A change in the SPH index of 0.2 translates to the additional consistent use of at least one sun protection behavior. Furthermore, Djaja et al [17] have shown using the item response theory that a change to “using a hat consistently” moves a person from above-average to below-average skin cancer risk [17].

In addition, personalized feedback information could be received using UVR-detecting dosimeters. The personal UV dosimeter provides feedback by sounding an alarm at a defined UVR threshold, alerting users the need for sun protection to reduce the risk of sunburn. Commercial interest has seen a large number of UVR-detecting devices being marketed directly toward the public. The devices can be worn as watches (attached to a strap) or pinned to clothing such as hats or shirts. There is a lack of evidence whether they aid consumers’ sun protection behaviors. To build the evidence for their efficacy for sun protection behavior change, the objective of this intervention trial was to evaluate one mobile phone app (SunSmart app, Cancer Council Victoria) and one personal UVR dosimeter monitor (Healthtronics SunSafe Pty Ltd), which has been shown in pretesting to provide accurate readings, and to assess the impact these have on young adults’ sun exposure and sun protection habits compared with a no intervention control group.

## Methods

### Study Design and Participants

**Figure 1:**
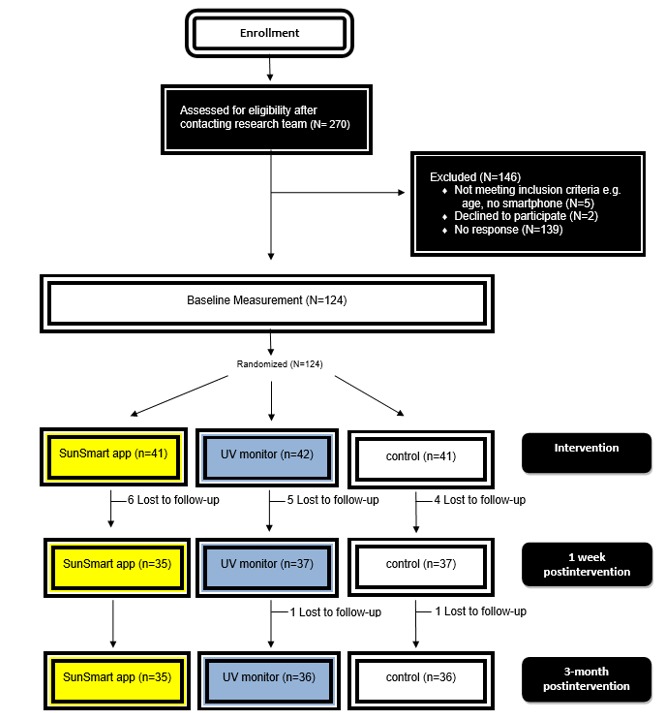
Flowchart of study recruitment. UV: ultraviolet.

The SknTec trial, conducted in Queensland, Australia, used a randomized controlled design with 2 intervention groups (SunSmart app or UVR monitor) and a measurement-only control group. Study approval was obtained from the Queensland University of Technology’s (QUT) Human Research Ethics Committee, and the study was conducted in accordance with the Declaration of Helsinki with written informed consent from all participants (approval number QUT 1400000302). This trial adheres to the Consolidated Standards of Reporting Trials EHEALTH for randomized controlled trials checklist (Multimedia Appendix 1). Eligibility criteria included young adults who were aged 18-35 years, had never been diagnosed with melanoma, had Fitzpatrick skin type 1-3, owned a smartphone, and were not a regular user of the SunSmart app or a personal UVR dosimeter. Participants (n=124) were recruited through Web via emails at the university or social media. Traditional media such as posters at sporting centers and in the local community were also used for recruitment (Figure 1). Prospective participants completed a screening telephone call or in-person visit at the university. The project was outlined, eligibility was determined, and written informed was consent obtained.

The data collection was performed using a staggered recruitment process during September-December 2015, with data collection running over Spring, Summer, and Autumn in Brisbane, Australia, when the UV index is consistently >6 and can reach 14+, requiring sun protection every day (Multimedia Appendix 2). UVR data recorded during the study were captured using the Australian Radiation Protection and Nuclear Safety Agency UV-Biometer model 501 detector (Solar Light Co, Philadelphia, PA, USA) located in Brisbane (latitude 27°S, 153°E). During the baseline 2-week period, participants completed a Web-based questionnaire and recorded their daily sun exposure, as well as physical activity, using a Web-based sun diary described previously [18]. After the baseline period, participants were block randomized (permuted blocks of 6), stratified by gender, using a computer-generated random number list created by the study software engineer independent from other study procedures. Participants were randomized at baseline when their allocation was created in the database. The research nurse and participants were blinded to the allocation of the intervention arms during the baseline phase, which was only revealed by the research nurse to participants at the commencement of the intervention phase.

**Figure 2:**
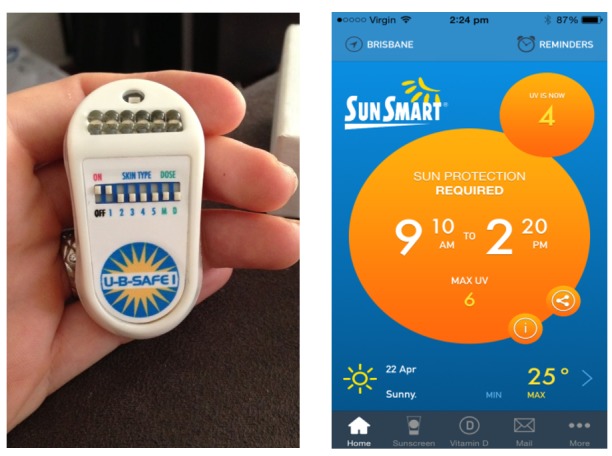
Intervention devices; left: personal ultraviolet radiation dosimeter monitor (Healthtronics SunSafe Pty Ltd, Australia); right: SunSmart app (Cancer Council Victoria, Australia).

For the 4-week intervention phase, participants were separated into 3 groups: (1) no intervention, measurement-only control group; (2) UVR monitor group, where participants were asked to wear a UVR dosimeter feedback device while outdoors (Healthtronics SunSafe Pty Ltd; Figure 2, image on left); or (3) the SunSmart app group, where participants were asked to download and use the free SunSmart phone app on their personal mobile phone (Cancer Council Victoria, Australia; Figure, image on right). During the 4-week intervention phase, participants were asked to complete daily sun diaries. Participants were emailed by the research team if the daily sun diaries had not been completed for >3 days in a row. At the completion of the intervention phase, participants in the UVR monitor group returned their monitors via mail. Participants in the SunSmart app group were emailed instructions to remove the app from their phone and asked to confirm it was uninstalled via return email.

Follow-up posttest measurements were taken at 1 week and 3 months postintervention, with participants completing a Web-based questionnaire and recording their sun exposure daily for 2 weeks using the Web-based sun diary. Of note, participants were reimbursed for their time at the end of the study with an Aus $70 gift card.

### Intervention Devices

#### Ultraviolet Radiation Monitor

The Healthtronics SunSafe UV dosimeter device (Figure 2) pins to an individual’s clothing and can be personalized for skin type alarming when UVR thresholds are met. This device computes a daily maximum UV dose for each particular skin type on a scale of 1-5 based on the Fitzpatrick skin types (1=very fair skin type, sunburns always, never suntans; 2=fair skin type, sunburns easily, minimal suntan; 3=light-medium skin type, some sunburn, gradual suntan; 4=medium skin type, minimally sunburns, always suntans; 5=medium-dark skin type, rarely sunburns, always suntan). Its UV detectors are housed on a sloping panel to adjust for the different positional orientation areas of the body exposed to the sun when standing up or lying down. The UV-B-SAFE 1 model is water resistant and solar powered with a charge time of 30 seconds in the sun. The UVR monitor plays a short tune when it is turned on to advise it has sufficient power. When the alarm sounds continuously, it means the user has reached his or her maximum UVR threshold for the day. The UV monitor is capable of providing real-time dose levels only and does not record personal UV exposure data to a Web-based database. All participants assigned to the UVR monitor device confirmed receiving the device, which contained the manufacturer’s printed instructions (Multimedia Appendix 3).

#### SunSmart App

The SunSmart app (Figure 2) displays the daily UV index, the weather for a range of Australian locations, and a daily time period when sun protection is required based on the most sun-sensitive skin type-1. During download, the app notification function was enabled to send daily reminders of the time periods sun protection is required. Furthermore, the SunSmart app gives recommendations for how people can best protect themselves from the sun, how the UV index works, and a vitamin D tracker and sunscreen calculator tool [19].

### Outcome Measures

Outcomes were assessed at baseline and 1 week and 3 months postintervention. Sociodemographic data were collected at baseline, including skin cancer risk factors (hair color, eye color, tendency to burn, ability to tan, personal or family history of skin excisions, or skin cancer) and sun protection attitudes and intentions using questionnaires previously developed [20]. An evaluation questionnaire was conducted at the end of the study and satisfaction with the intervention devices and the intervention delivery was assessed. Participants were asked to rate on a 10-point Likert scale how satisfied they were with the intervention device (1=not at all satisfied, 5=moderately satisfied, 10=extremely satisfied). Participants were asked to self-report their use of the intervention devices.

#### Sun Protection Habits Index

The primary outcome measure was the SPH index developed by Glanz et al [21] measured at baseline and evaluation time points. It queries the frequency of 6 sun-protective methods that are used when outdoors using a 4-point Likert scale (1=never or rarely to 4=always), which are averaged to derive the score, including wearing a shirt with sleeves, wearing a hat, wearing sunglasses, using sunscreen, staying in the shade, and limiting time in the sun during midday hours. SPH index test-retest reliability has good internal consistency (0.76) and test-retest reliability (0.78), and estimates of its validity have been previously reported [22].

#### Ultraviolet Radiation Exposure, Sunburn, and Physical Activity

Data on frequency of sunburn and suntan (number of times), time spent in the sun unprotected on weekdays and weekends (minutes and hours, body areas exposed unprotected), sunscreen use (yes or no), and physical activity (minutes and hours) were collected using the Web-based sun diary [18].

### Statistical Analysis

Generalized estimating equations (GEE) models were used for analyzing changes in the mean combined SPH index over time and for individual items, including wearing a shirt with sleeves, sunglasses, staying in the shade, sunscreen use, limiting time in the sun, and wearing a hat. The model contained the group, gender, and skin type. In addition, we fitted the interaction of time with (1) gender and (2) group. The sun diary variables (UVR unprotected exposure, UVR unprotected torso exposure, sunscreen use, and physical activity) were dichotomized into (1) yes (improvement of ≥5 minutes from every individual’s baseline) or (2) no (no improvement or improvement of <5 minutes). Number of participants in each category and percentage are presented in Table 1. Furthermore, logistic binary regression analyses were used to detect the odds of improvement in each intervention group compared with the control group.

Regarding sample size calculations, to detect an effect size of 0.4, which allows a change in the mean SPH index of 0.2 (from a mean score of 2.3-2.5 at follow-up), given a common SD of 0.5 among 3 groups where 2 of the groups are compared with one control group (based on the Dunnett multiple comparison test), an optimal sample size of 300 was determined. However, only 55% of the requested funding was received, allowing us to recruit a maximum of 200 participants; of them, 124 completed the study, allowing the ability to detect an effect size of 0.6, or a 0.3 difference in SPH among groups [23].

**Table 1:** Participant characteristics.

Characteristics		Total (n=124)	Control (n=41)	SunSmart App (n=41)	Ultraviolet radiation monitor (n=42)
Mean age in years		25.8	25.4	26.5	25.4
**Gender, n (%)**					
	Female	85 (68.5)	26 (63.4)	32 (78.0)	27 (64.3)
	Male	39 (31.5)	15 (36.6)	9 (22.0)	15 (35.7)
**Highest completed education, n (%)**					
	Completed high school	21 (16.9)	8 (19.5)	7 (17.1)	6 (14.3)
	Trade or technical certificate or diploma	12 (9.7)	6 (14.6)	3 (7.3)	3 (7.1)
	University or college degree	91 (73.4)	27 (65.9)	31 (75.6)	33 (78.6)
**Current work situation, n (%)**					
	Employed full-time	24 (19.4)	9 (22.0)	9 (22.0)	6 (14.3)
	Part-time or casual	20 (16.1)	6 (14.6)	8 (19.5)	6 (14.3)
	Student	80 (64.5)	26 (63.4)	24 (58.5)	30 (71.4)
**Is your main job now…, n (%)**					
	Mainly indoors	109 (87.9)	38 (92.7)	35 (85.4)	36 (85.7)
	Mainly outdoors	2 (1.6)	N/A^a^	1 (2.4)	1 (2.4)
	About equal amounts indoors and outdoors	13 (10.5)	3 (7.3)	5 (12.2)	5 (11.9)
**Born in Australia, n (%)**					
	Yes	58 (46.8)	20 (48.8)	20 (48.8)	18 (42.9)
	No	66 (53.2)	21 (51.2)	21 (51.2)	24 (57.1)
**Eye color, n (%)**					
	Blue or gray	28 (22.6)	9 (22.0)	13 (31.7)	6 (14.2)
	Green	16 (12.9)	5 (12.2)	4 (9.8)	7 (16.7)
	Brown	65 (52.4)	24 (58.5)	19 (46.3)	22 (52.4)
	Other^b^	15 (12.1)	3 (7.3)	5 (12.2)	7 (16.7)
**Natural hair color at the age of 21 years (or now if younger), n (%)**					
	Red (including auburn)	7 (5.6)	3 (7.3)	2 (4.9)	2 (4.8)
	Fair or blonde (including white)	8 (6.4)	1 (2.5)	4 (9.7)	3 (7.1)
	Light brown	25 (20.2)	8 (19.5)	10 (24.4)	7 (16.7)
	Dark brown	42 (33.9)	16 (39.0)	10 (24.4)	16 (38.1)
	Black	42 (33.9)	13 (31.7)	15 (36.6)	14 (33.3)
**Skin color, n (%)**					
	Fair	72 (58.1)	24 (58.6)	21 (51.2)	27 (64.3)
	Medium	38 (30.6)	13 (31.7)	15 (36.6)	10 (23.8)
	Olive or dark	13 (10.5)	3 (7.3)	5 (12.2)	5 (11.9)
	Black	1 (0.8)	1 (2.4)	N/A	N/A
**Skin reaction in strong summer sun for 30 minutes without protection, n (%)**					
	My skin would not burn at all	10 (8.1)	5 (12.2)	1 (2.5)	4 (9.5)
	My skin would burn lightly	44 (35.5)	13 (31.7)	13 (31.7)	18 (42.9)
	My skin would burn moderately	47 (37.9)	15 (36.6)	16 (39.0)	16 (38.1)
	My skin would burn severely	23 (18.5)	8 (19.5)	11 (26.8)	4 (9.5)
**Skin reaction if you spend several weeks at the beach and you are often in the strong sun, without any protection, n (%)**					
	My skin would not tan	10 (8.0)	4 (9.8)	2 (4.9)	4 (9.5)
	My skin would tan lightly	25 (20.2)	8 (19.5)	8 (19.5)	9 (21.5)
	My skin would tan moderately	60 (48.4)	21 (51.2)	18 (43.9)	21 (50.0)
	My skin would tan deeply	29 (23.4)	8 (19.5)	13 (31.7)	8 (19.0)
**Previous skin cancer, mole, or other spot(s) removed or treated, n (%)**					
	Yes	15 (12.1)	5 (12.2)	7 (17.1)	3 (7.1)
	No	108 (87.1)	36 (87.8)	34 (82.9)	38 (90.5)
	Unsure or do not know	1 (0.8)	N/A	N/A	1 (2.4)

^a^N/A: not applicable.

^b^Other: mixed or undefined eye color.

## Results

### Participant Characteristics

The mean age of all participants was 25.8 (range 18-35) years and 68.5% (85/124) were females; 73.3% (91/124) participants had a university degree, and 87.9% (109/124) of participants worked mainly indoors (Table 1). More than half of the participants (72/124, 58.1%) had fair skin that was sun sensitive, including skin that moderately or severely burns after 30 minutes of sun exposure in summer without protection. The characteristics were quite evenly distributed among the groups.

### Intervention and Data Collection Completeness

In the UVR monitor group, 94% (34/36) of participants self-reported using the UVR monitor all or some of the time when outside. All participants in the UVR monitor group confirmed receiving the device and returned the UVR monitor postintervention. All SunSmart app group participants downloaded the app on their smartphone, and 97% (34/35) of participants reported they received the daily UV index sun protection pop-up notifications. All participants completed the baseline questionnaire (n=124); 87.9% (109/124) completed the 1-week and 86.3% (107/124) completed the 3-month postintervention questionnaire. The Web-based sun diary was completed by 95.2% (118/124) participants at baseline, 88.7% (110/124) participants during the intervention and 1 week postintervention, and 86.3% (107/124) participants at 3 months postintervention.

### Sun Protection Habits Index

At baseline, the mean SPH index value was 2.42 (SE 0.08) for the control group, 2.36 (SE 0.08) for the UV monitor group, and 2.47 (SE 0.07) for the SunSmart app group (Multimedia Appendix 4). At the 3-month time point, the SPH index had improved by +0.13, +0.14, and +0.06 in the UV monitor, SunSmart app, and control groups, respectively (*P*=.001). This increase did not differ significantly by group, resulting in a nonsignificant group by time interaction (*P=*.35, GEE).

### Ultraviolet Radiation Exposure, Sunscreen Use, and Physical Activity

Compared with the control group, a significantly greater proportion of the UVR monitor group participants improved their sun protection on weekends during the intervention phase (OR 2.706, 95% CI 1.047-6.992, *P*=.04; Table 2). This reduction in weekend unprotected exposure in the UVR monitor group was on average 58.78 (SE 13.41) minutes each day, a 58.5% (SE 5.18) reduction from baseline exposure in those who improved (n=23). The UVR monitor group continued demonstrating an improvement in sun protection on weekends 3 months postintervention (OR 3.130, 95% CI 1.196-8.190, *P*=.02; Table 2). This reduction in weekend unprotected exposure was on average 61.05 (SE 12.51) minutes each day, a 75.3% (SE 5.66) reduction from baseline exposure in those who improved (n=23). Weekend UVR exposure did not differ significantly between the control and the SunSmart app groups at any time point. Total and weekday UVR exposure did not differ significantly at any time point between the intervention group and the control group. Sunscreen use and physical activity levels remained largely unchanged across the study period in all 3 groups (Multimedia Appendix 4). The number of weekdays that sunscreen was used was observed in the SunSmart app group 1 week postintervention, but these results were not statistically significant (OR 2.808, 95% CI 0.854-9.238, *P*=.09; Multimedia Appendix 4, Supplementary Table 2), which was an increase in sunscreen use from 3 out of 10 days at baseline compared with 4 out of 10 days at 1 week postintervention.

### Unprotected Ultraviolet Radiation Torso Exposure Incidence

We observed that 52.3% (56/107) of participants who completed the study reported unprotected UVR torso exposure at one or more time points during the study. Unprotected UVR torso exposure did not differ by gender, with 55% (41/75) of females and 47% (15/32) of males in the study. Unprotected torso exposure incidence did not differ significantly between the control and intervention groups at any time point (Multimedia Appendix 4).

**Table 2:** Participants who reduced their time in the sun unprotected.

Characteristics			Yes^a^, n (%)	No, n (%)	OR	95% CI	*P* value
**During intervention^b^**							
	**Weekday and weekend ultraviolet radiation (UVR) unprotected exposure**						
		Control	14 (38.9)	22 (61.1)	1.00	N/A^c^	Ref^d^
		SunSmart app	16 (44.4)	20 (55.6)	1.379	0.525-3.627	.51
		UVR monitor	18 (47.4)	20 (52.6)	1.407	0.557-3.549	.47
	**Weekday UVR unprotected exposure**						
		Control	15 (41.7)	21 (58.3)	1.00	N/A	Ref
		SunSmart app	11 (30.6)	25 (69.4)	0.622	0.231-1.670	.35
		UVR monitor	18 (47.4)	20 (52.6)	1.280	0.508-3.225	.60
	**WeekEnd UVR unprotected exposure**						
		Control	13 (36.1)	23 (63.9)	1.00	N/A	Ref
		SunSmart app	15 (41.7)	21 (58.3)	1.533	0.563-4.176	.40
		UVR monitor	23 (60.5)	15 (39.5)	2.706	1.047-6.992	.04
**1 week after the intervention^e^**							
	**Weekday and weekend UVR unprotected exposure**						
		Control	18 (48.6)	19 (51.4)	1.00	N/A	Ref
		SunSmart app	21 (58.3)	15 (41.7)	1.582	0.611-4.095	.35
		UVR monitor	20 (54.1)	17 (45.9)	1.242	0.498-3.095	.64
	**Weekday UVR unprotected exposure**						
		Control	19 (51.4)	18 (48.6)	1.00	N/A	Ref
		SunSmart app	17 (47.2)	19 (52.8)	0.972	0.375-2.514	.95
		UVR monitor	14 (37.8)	23 (62.2)	0.576	0.228-1.455	.24
	**Weekend UVR unprotected exposure**						
		Control	14 (37.8)	23 (62.2)	1.00	N/A	Ref
		SunSmart app	19 (52.8)	17 (47.2)	2.238	0.831-6.023	.11
		UVR monitor	21 (56.8)	16 (43.2)	2.173	0.853-5.535	.10
**3 months after the intervention^f^**							
	**Weekday and weekend UVR unprotected exposure**						
		Control	16 (44.4)	20 (55.6)	1.00	N/A	Ref
		SunSmart app	20 (57.1)	15 (42.9)	1.748	0.666-4.587	.26
		UVR monitor	18 (50.0)	18 (50.0)	1.250	0.495-3.162	.64
	**Weekday UVR unprotected exposure**						
		Control	14 (38.9)	22 (61.1)	1.00	N/A	Ref
		SunSmart app	15 (42.9)	20 (57.1)	1.205	0.456-3.182	.71
		UVR monitor	14 (38.9)	22 (61.1)	1.000	0.385-2.595	.99
	**Weekend UVR unprotected exposure**						
		Control	13 (36.1)	23 (63.9)	1.00	N/A	Ref
		SunSmart app	18 (51.4)	17 (48.6)	1.898	0.717-5.026	.20
		UVR monitor	23 (63.9)	13 (36.1)	3.130	1.196-8.190	.02

^a^Yes: reduced average daily minutes of time unprotected in the sun compared with baseline based on the self-reported diary entry.

^b^n=110 for the intervention measurement period.

^c^N/A: not applicable.

^d^Ref: Reference *P* value.

^e^n=109 for the 1 week after intervention measurement period.

^f^n=107 for the 3 months after intervention measurement period.

### Sunburn and Suntan Incidence

Sunburn rates were high with 58.0% (62/107) of participants reporting one or more (range 1-11) sunburns during the study. In this study, 63% (46/75) of all females and 50% (16/32) of all males reported one or more sunburns. No differences were observed between groups in the incidence of sunburn and suntan (Multimedia Appendix 4). Deliberate suntanning behavior during the study was reported by 29.9% (32/107) of participants (range 1-7), which was observed in 37% (28/75) of females and 13% (4/32) of males. Sunburn and suntan incidence did not differ significantly between the control and intervention groups at any time point (Multimedia Appendix 4).

### Satisfaction With Intervention Devices

Two-thirds of participants (UVR monitor group, 23/36, 64%; SunSmart app group, 23/35, 66%) found their intervention helpful to guide their sun-protective behavior (Multimedia Appendix 4). About half of the participants (UVR monitor group, 17/36, 47%; SunSmart app group, 19/35, 54%) self-reported that they changed or modified their behavior in response to the output from the device. In the UVR monitor group, 47% (17/36) participants found the device to be encouraging to engage in sun protection; however, only 19% (7/36) would purchase one. In the SunSmart app group, 63% (22/35) of participants found the app encouraging to engage in sun protection, and 40% (14/35) would download it in the future. In addition, 36% (13/36) of participants in the UVR monitor group and 60% (21/35) in the SunSmart app group reported the device repeated what they already knew. UVR monitor participants’ mean response for intervention device satisfaction out of a scale from 1 to 10 was 5.19 (SE 0.47), and the SunSmart app participants’ mean response was 5.66 (SE 0.36). Qualitative feedback from the open-ended responses was grouped by themes, showing that young adults found the SunSmart app likable and easy to navigate. However, feedback for the content was that “it never changes,” “it’s always 8 am to 4 pm use sun protection,” and “gets boring.” Participants liked the personalized feedback by the UVR monitor; however, they were unlikely to carry a separate device for UVR detection. Participants wanted more control over the feedback method that the UVR monitor provides and would find it more appealing if they could tailor the alert or alarm for their specific preferences (eg, if it allowed the users to select their song as an alarm or a more subtle vibration alert).

## Discussion

### Principal Findings

This randomized controlled trial (RCT) examined the impact of using digital or electronic technologies to improve young peoples’ sun protection or sun exposure behaviors. We found no consistent benefit of providing participants with either a mobile phone app or electronic dosimeter for their sun protection habits compared with a no intervention control group. Despite all 3 groups reporting a similar improvement in the main outcome measure, the SPH index, our young participants continued to experience high sunburn rates throughout the study period.

While we did not observe a significant effect on the SPH index, some differences in specific measures of sun exposure were noted among the groups. The reduction in weekend unprotected sun exposure for the UVR monitor group was encouraging as weekend sun exposure is common, with 21% of adolescents reporting being sunburnt on an average summer weekend in Australia [3]. Previous studies have suggested a suboptimal understanding of the UV index and peak UVR times among young adults as reasons for this [24,25]. Our findings suggest using a personal UV monitor that produces an auditory alarm may be helpful to educate young adults on what is a safe level of UVR exposure for their skin type. However, this was not enough to also change their sun protection habitual behavior as measured by the SPH index over and above the change achieved in the control group. Moreover, the change was not strong enough to reduce sunburn rates. Participants commented that they would have liked to further personalize the alarm sounds, which could be tested in future studies. Furthermore, carrying a separate device for UVR detection was mentioned as being burdensome by some participants, and future work could explore the potential to utilize movement and light sensors in smart devices already carried by people as a way of capturing UVR exposure.

Previous studies testing UVR monitors have reported varying results. Carli et al [26] (n=91) found longer sun exposure (*P*=.003) and more frequent sunburns (*P*=.004) in the UV monitor group compared with the control group. These unfavorable adverse outcomes may have been due to limitations of the UV monitor’s detector (SunCast UV monitor), which when not ideally positioned toward the sun may have underreported UVR exposure. The UVR monitor (UV-B-Safe model) tested in our study had a sloping panel of detectors to better accommodate positional body orientations and alerted users with an auditory alarm in contrast to the SunCast UV monitor, which displays the UVR measurement on a UV index scale and did not have an alarm function. A Swedish study tested a UVR intensity indicator (Teraco, Inc, USA), which changed color if the UVR levels were moderate, high, or extreme [27]. These authors reported no statistically significant differences in the frequency of sunbathing, sunburn, or attitudes toward being in the sun between groups receiving either the UVR monitor or written information about sun protection in those aged 18-37 years. However, participants’ use of the UVR intensity indicator device was low, with only 42% using it compared with 94% (34/36) of participants in this study.

In a randomized clinical trial evaluating the Solar Cell app, which provided personalized, real-time sun protection advice and alerts, it was found that a greater proportion of app users *reduced* the use of sunscreen (29% vs 35%) in the control group (*P*=.048), with a greater proportion of app users increasing the use of shade instead (41% vs 34% control; *P*=.03) [15]. Findings from our study and a study by Buller et al [15] showed no difference in the number of sunburns between app users and control group participants. Of note, 57.9% (62/107) of participants reported one or more sunburns during this study, which is slightly higher than the 47% rate observed among young adults in the United States by Buller et al [28]. The prevalence of sunburn among young men was not significantly different from the prevalence among young women, consistent with previous reports from the United States [28]. High-risk sun exposure behaviors such as torso exposure were commonly reported, and suntanning behaviors were similar to those reported in previous studies, with a higher proportion of females reporting suntanning compared with males [29].

Participants provided a satisfaction rating score of 5 out of 10, indicating they were moderately satisfied with the intervention devices, and the qualitative feedback received demonstrated that personalized, tailored engaging feedback is preferred by young adults. They recommended combining elements from both intervention devices, which may be advantageous to reduce sunburn rates in this population. In addition, Buller et al [15] reported the beneficial impact of tailoring information to each user “in the moment,” promoting a sense of volition, choice, and control. Likewise, our previous work provides evidence for the benefit of personalized approaches. The Healthy Text study recruited 574 participants (age, 18-42 years) who received 21 motivational messages on sun protection compared with physical activity attention control messages [20]. At 12 months postrandomization, the sun protection group had significantly greater improvement in its SPH index than the control group [16], and building behavioral capacity (eg, obtaining information and receiving reminders) was the most valued aspect of the messages [30]. Heckman et al [31] recently reported significant decreases in UV exposure and increases in SPH index 3 and 12 weeks after baseline for participants who received a tailored multimedia internet intervention program (UV4.me). These studies have illustrated that further improvements to the technology platforms are needed to reduce the sunburn prevalence in young adult populations.

The observation of improvement in the measurement-only control group may be due to participants completing multiple surveys about sun protection, which may have increased attention to their own sun-protective behavior. Campbell and Stanley [32] described the impact of exposure to a pretest or intervening assessment influences performance on a posttest, and a more recent work by Koster et al [33] reported that simply keeping a UV exposure diary increased attention toward the behavior examined. The change in the control group could also be explained by the Hawthorne effect, which is when a person’s behavior changes because he or she is knowingly under observation. Future study design could incorporate an attention control group that receives equivalent information about an alternative activity, for example, physical activity. Other reasons the control group improved could be exposure to other sun protection programs implemented over the study period (not measured) or seasonal variation with baseline data collected in Spring and follow-up data collected at the end of Summer. Seasonal variations may be partly responsible for some of the changes observed; for example, if young people were more likely to use sunscreen later in the summer or get sunburnt in early summer, or be employed seasonally with changing work schedules.

The strengths of this study include the RCT design, which created equivalent groups, high participant retention, and testing relatively low-cost and readily available intervention components. Limitations of this study include self-reported outcome measures, which are subject to recall and social desirability biases. The proper use of sun protection methods (eg, the adequate thickness in the application of sunscreen) was not objectively assessed. A further limitation is that UV monitor compliance was self-reported and personal UV exposure data were not captured in a Web-based study database. Our sample size was relatively small and may have led to low statistical power, contributing to the nonsignificant findings. We used convenience sampling in a university setting, and participation also involved time-intensive activities including completing a screening questionnaire and daily sun diaries, which may be perceived as too burdensome, leading to a unique sample. Furthermore, participants were mainly highly educated (university or college degree) and worked indoors, and results may not be generalizable to other subgroups of the population.

### Conclusions

We aimed to provide evidence for the effectiveness of digital and mobile technologies to improve sun protection behaviors among young adults. Self-monitoring devices for maintaining wellness are becoming more widespread. Tracking one’s health may enable consumers to improve health outcomes, but we found a relatively limited impact on important sun protection behaviors in this young population. Hence, an even more personalized approach to public health efforts may be needed to facilitate UVR protection and avoid increases in skin cancer cases.

## Appendix

Multimedia Appendix 1 CONSORT‐EHEALTH checklist (V 1.6.1).

Multimedia Appendix 2 Supplementary Figure 1.

Multimedia Appendix 3 Supplementary Figure 2.

Multimedia Appendix 4 Supplementary Tables 1-7.

## Abbreviations

GEE: generalized estimating equations
OR: odds ratio
QUT: Queensland University of Technology
RCT: randomized controlled trial
SPH: Sun protection habits index
UVR: ultraviolet radiation
