# Supplementary material for: A Mobile Technology Intervention With Ultraviolet Radiation Dosimeters and Smartphone Apps for Skin Cancer Prevention in Young Adults: Randomized Controlled Trial
Source: JMIR Mhealth Uhealth. 2018 Nov 28;6(11):e199. doi: 10.2196/mhealth.9854 (PMC6291679; doi:10.2196/mhealth.9854)
Supplement: Multimedia Appendix 2 [file mhealth_v6i11e199_app2.pdf]

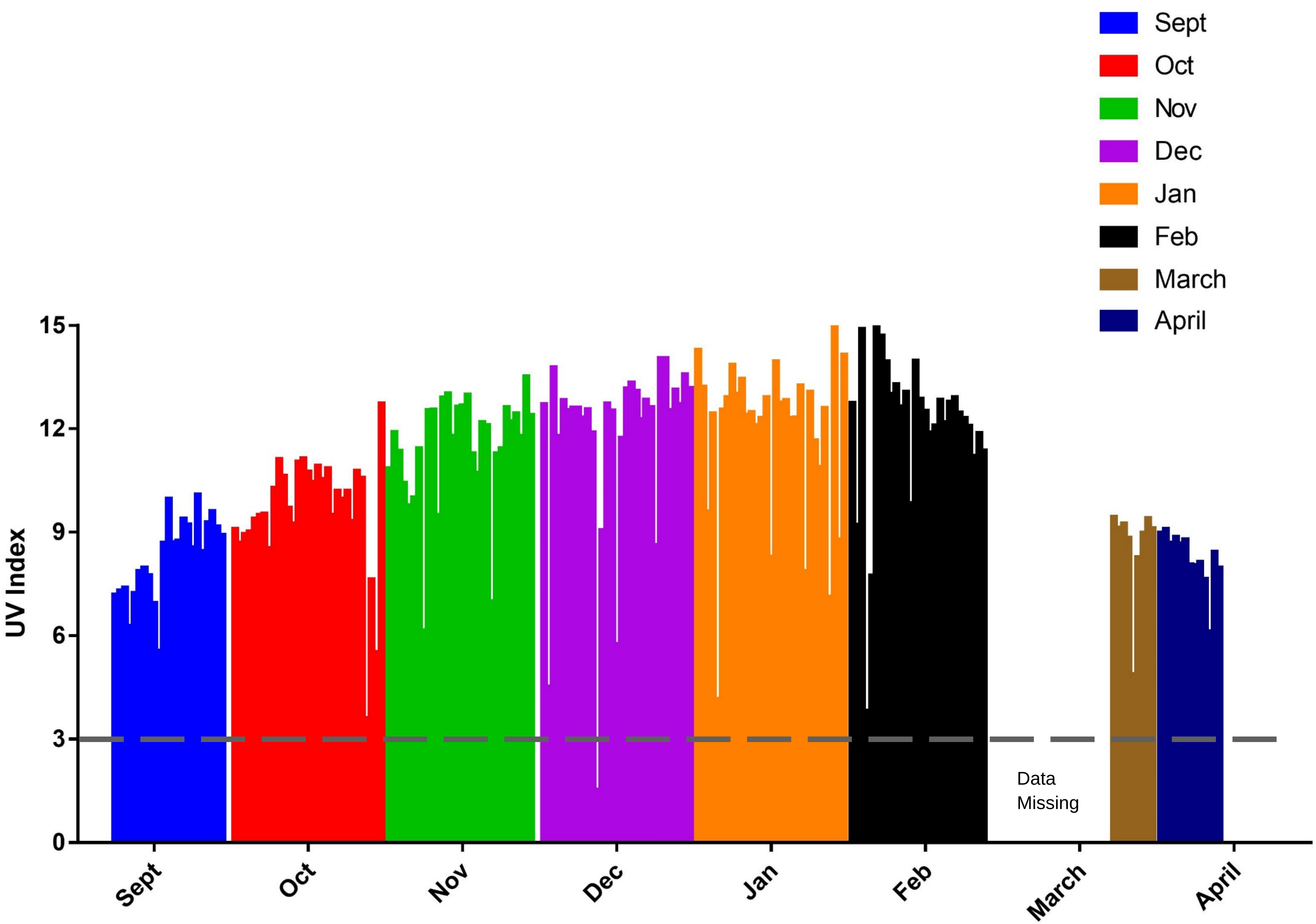

Figure S1 UV index Data during the Study Period. The daily peak UV level recorded using a UV-Biometer model 501 detector (Solar Light Co, Philadelphia, PA) and data displayed using the UV index scale. The UVR data was captured by the Australian Radiation Protection and Nuclear Safety Agency detector (Brisbane, latitude 27ºS, 153 ºE). Data was available each day of the study from (8th Sept, 2015 to 13th April, 2016) with data missing during a maintenance period between Feb 29th-Mar 22rd.
