# Supplementary material for: A Mobile Technology Intervention With Ultraviolet Radiation Dosimeters and Smartphone Apps for Skin Cancer Prevention in Young Adults: Randomized Controlled Trial
Source: JMIR Mhealth Uhealth. 2018 Nov 28;6(11):e199. doi: 10.2196/mhealth.9854 (PMC6291679; doi:10.2196/mhealth.9854)
Supplement: Multimedia Appendix 3 [file mhealth_v6i11e199_app3.pdf]

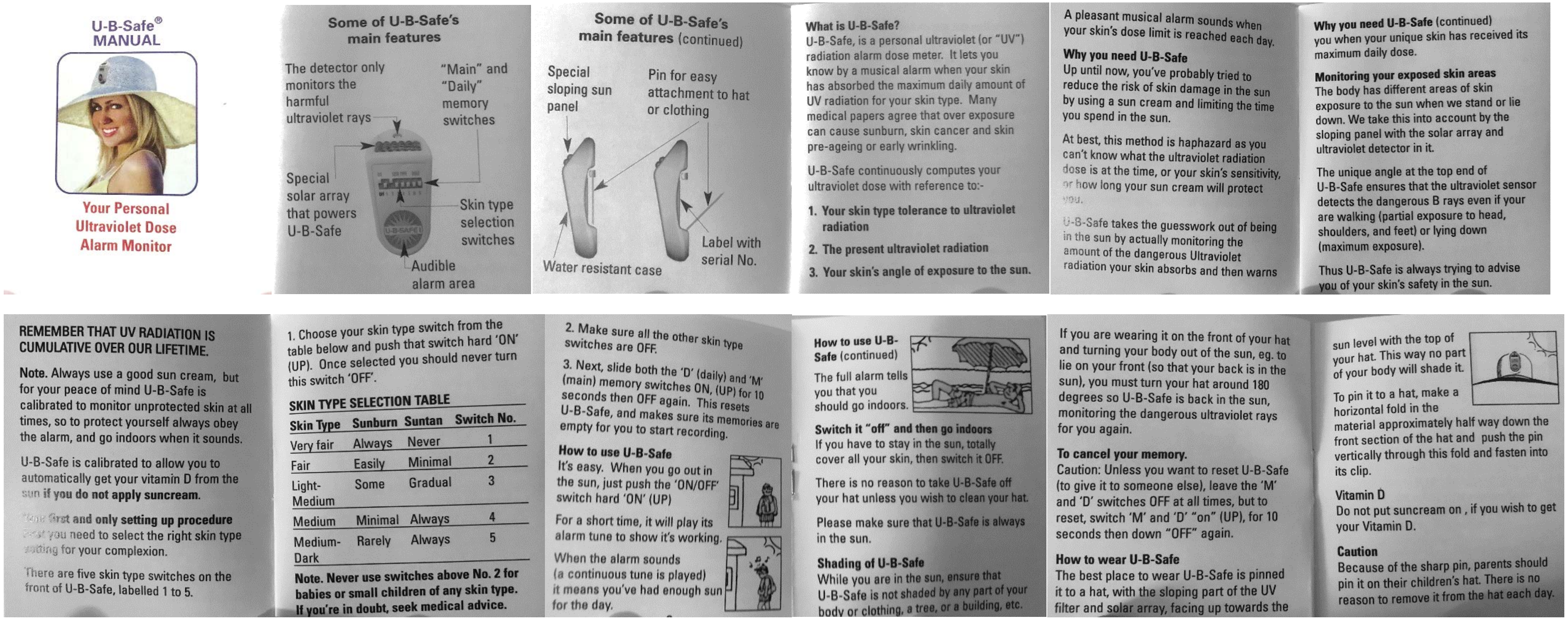

U-B-Safe®
MANUAL

Your Personal Ultraviolet Dose Alarm Monitor

Some of U-B-Safe's main features

The detector only monitors the harmful ultraviolet rays

"Main" and "Daily" memory switches

Special solar array that powers U-B-Safe

Skin type selection switches

Audible alarm area

Some of U-B-Safe's main features (continued)

Special sloping sun panel

Pin for easy attachment to hat or clothing

Label with serial No.

Water resistant case

What is U-B-Safe?
U-B-Safe, is a personal ultraviolet (or "UV") radiation alarm dose meter. It lets you know by a musical alarm when your skin has absorbed the maximum daily amount of UV radiation for your skin type. Many medical papers agree that over exposure can cause sunburn, skin cancer and skin pre-ageing or early wrinkling.

U-B-Safe continuously computes your ultraviolet dose with reference to:-

1. **Your skin type tolerance to ultraviolet radiation**
2. **The present ultraviolet radiation**
3. **Your skin's angle of exposure to the sun.**

A pleasant musical alarm sounds when your skin's dose limit is reached each day.

Why you need U-B-Safe
Up until now, you've probably tried to reduce the risk of skin damage in the sun by using a sun cream and limiting the time you spend in the sun.

At best, this method is haphazard as you can't know what the ultraviolet radiation dose is at the time, or your skin's sensitivity, or how long your sun cream will protect you.

U-B-Safe takes the guesswork out of being in the sun by actually monitoring the amount of the dangerous Ultraviolet radiation your skin absorbs and then warns

Why you need U-B-Safe (continued)
you when your unique skin has received its maximum daily dose.

Monitoring your exposed skin areas
The body has different areas of skin exposure to the sun when we stand or lie down. We take this into account by the sloping panel with the solar array and ultraviolet detector in it.

The unique angle at the top end of U-B-Safe ensures that the ultraviolet sensor detects the dangerous B rays even if your are walking (partial exposure to head, shoulders, and feet) or lying down (maximum exposure).

Thus U-B-Safe is always trying to advise you of your skin's safety in the sun.

REMEMBER THAT UV RADIATION IS CUMULATIVE OVER OUR LIFETIME.

Note. Always use a good sun cream, but for your peace of mind U-B-Safe is calibrated to monitor unprotected skin at all times, so to protect yourself always obey the alarm, and go indoors when it sounds.

U-B-Safe is calibrated to allow you to automatically get your vitamin D from the sun **if you do not apply suncream.**

Your first and only setting up procedure
First you need to select the right skin type setting for your complexion.

There are five skin type switches on the front of U-B-Safe, labelled 1 to 5.

1. Choose your skin type switch from the table below and push that switch hard 'ON' (UP). Once selected you should never turn this switch 'OFF'.

SKIN TYPE SELECTION TABLE

Skin Type	Sunburn	Suntan	Switch No.
Very fair	Always	Never	1
Fair	Easily	Minimal	2
Light-Medium	Some	Gradual	3
Medium	Minimal	Always	4
Medium-Dark	Rarely	Always	5

Note. Never use switches above No. 2 for babies or small children of any skin type. If you're in doubt, seek medical advice.

2. Make sure all the other skin type switches are OFF.

3. Next, slide both the 'D' (daily) and 'M' (main) memory switches ON, (UP) for 10 seconds then OFF again. This resets U-B-Safe, and makes sure its memories are empty for you to start recording.

How to use U-B-Safe
It's easy. When you go out in the sun, just push the 'ON/OFF' switch hard 'ON' (UP)

For a short time, it will play its alarm tune to show it's working.

When the alarm sounds (a continuous tune is played) it means you've had enough sun for the day.

How to use U-B-Safe (continued)
The full alarm tells you that you should go indoors.

Switch it "off" and then go indoors
If you have to stay in the sun, totally cover all your skin, then switch it OFF.

There is no reason to take U-B-Safe off your hat unless you wish to clean your hat.

Please make sure that U-B-Safe is always in the sun.

Shading of U-B-Safe
While you are in the sun, ensure that U-B-Safe is not shaded by any part of your body or clothing, a tree, or a building, etc.

If you are wearing it on the front of your hat and lie on your front (so that your back is in the sun), you must turn your hat around 180 degrees so U-B-Safe is back in the sun, monitoring the dangerous ultraviolet rays for you again.

To cancel your memory.
Caution: Unless you want to reset U-B-Safe (to give it to someone else), leave the 'M' and 'D' switches OFF at all times, but to reset, switch 'M' and 'D' "on" (UP), for 10 seconds then down "OFF" again.

How to wear U-B-Safe
The best place to wear U-B-Safe is pinned it to a hat, with the sloping part of the UV filter and solar array, facing up towards the

sun level with the top of your hat. This way no part of your body will shade it.

To pin it to a hat, make a horizontal fold in the material approximately half way down the front section of the hat and push the pin vertically through this fold and fasten into its clip.

Vitamin D
Do not put suncream on , if you wish to get your Vitamin D.

Caution
Because of the sharp pin, parents should pin it on their children's hat. There is no reason to remove it from the hat each day.

Caution (continued)
Please check with your health professional if you are under medication or on a special diet, to ensure that it will not affect your skin in the sun.

An aid to better hearing – U-B-Safe 2
If you are a little hard of hearing due to a noisy environment, etc then perhaps the U-B-Safe 2 monitor would suit you better. It has an ear piece for easier hearing. The U-B-Safe 2 costs a little more but can be exchanged for your U-B-Safe 1 plus a small additional fee.

Hard hat attachment kit - U-B-Safe 3
A special hard hat attachment kit is also available from your retailer or from the manufacture's address or website at the end of this manual.

Please remember
Our skin is one of the largest organs of the body, and because it is our outside protection it can easily be abused by the environment.

The more damage we do to our skin the more it costs in cosmetics and medical treatment. As our immune system declines with age previous sun excesses can come back as skin cancers, melanomas, etc.

U-B-Safe important notes
Don't let anything obstruct the round ultraviolet detector at the top of U-B-Safe.

Clean the UV filter every day. Use a soft clean cloth or tissue. Do not use harsh, abrasive cleansers – use window or lens cleaner on a cotton bud.

U-B-Safe important notes (continued)
Always respect the power of the noon-day sun in summer and try to avoid going out between 10.00 am and 3.30 pm.

Always wear adequate sun-blocking clothing, a wide brimmed hat, high quality sun glasses, and a good sun cream.

Please remember a healthy skin only needs a little sun.

Children should not play with U-B-Safe as it has a sharp pin and they could also put dirt on the detector area causing it to malfunction.

Note: U-B-Safe is not designed for use in solarium tanning units.

PRODUCT DISCLAIMER
Healthtronics Sunsafe Pty Ltd (HSP) states, that U-B-Safe has been developed as a scientific instrument and not a medical device and can only give general information on sun exposure if used in accordance with these instructions and will not give useful information if used inappropriately. HSP is not responsible for a user's wrong skin selection.

HSP makes no warranties expressed or implied about the effectiveness of the Product in preventing any skin disease, sunburn, melanomas or skin cancer.

HSP shall be under no liability whatsoever for damages, direct or consequential, arising out of the use of the Product.

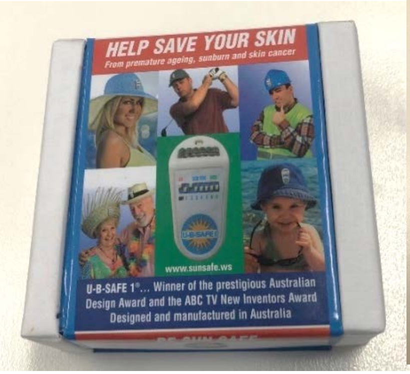

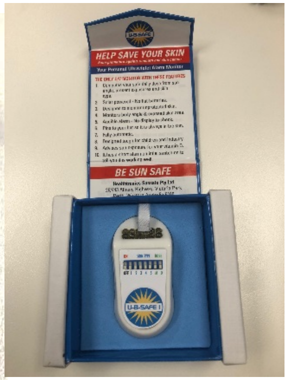

Figure S2 Instruction Booklet and labelling details for the UV monitor device (Healthtronics SunSafe Pty Ltd, Australia)
