# Supplementary material for: A Mobile Technology Intervention With Ultraviolet Radiation Dosimeters and Smartphone Apps for Skin Cancer Prevention in Young Adults: Randomized Controlled Trial
Source: JMIR Mhealth Uhealth. 2018 Nov 28;6(11):e199. doi: 10.2196/mhealth.9854 (PMC6291679; doi:10.2196/mhealth.9854)
Supplement: Multimedia Appendix 4 [file mhealth_v6i11e199_app4.pdf]

Table S1 Mean Sun Protection Habits

Sun Protection Habits (SPH)	Baseline n=124 Mean[a] (SE)	1 week after the intervention n=109 Mean* (SE)	3 months after the intervention n=107 Mean* (SE)
Wear a long shirt with sleeves			
Control	2.94 (0.14)	2.95 (0.14)	2.84 (0.14)
SunSmart App	3.04 (0.15)	2.73 (0.16)	2.89 (0.16)
UVR monitor	3.06 (0.16)	2.81 (0.18)	2.69 (0.16)
Wear Sunglasses			
Control	2.34 (0.13)	2.24 (0.14)	2.67 (0.12)
SunSmart App	2.18 (0.17)	2.13 (0.18)	2.48 (0.15)
UVR monitor	2.11 (0.19)	2.34 (0.18)	2.29 (0.19)
Stay in the shade			
Control	2.80 (0.10)	2.92 (0.10)	2.97 (0.10)
SunSmart App	2.87 (0.13)	2.86 (0.12)	3.04 (0.11)
UVR monitor	2.71 (0.11)	2.86 (0.13)	3.04 (0.12)
Use sunscreen			
Control	2.12 (0.12)	1.88 (0.11)	2.01 (0.10)
SunSmart App	2.01 (0.15)	2.18 (0.15)	2.22 (0.15)
UVR monitor	2.03 (0.16)	2.20 (0.18)	2.26 (0.17)
Limit your time in the sun during midday hours			
Control	2.67 (0.14)	2.83 (0.14)	2.77 (0.13)
SunSmart App	2.83 (0.15)	2.84 (0.15)	2.99 (0.14)
UVR monitor	2.72 (0.14)	2.86 (0.17)	2.98 (0.14)
Wear a hat			
Control	1.61 (0.11)	1.32 (0.10)	1.66 (0.11)
SunSmart App	1.83 (0.15)	1.71 (0.14)	2.06 (0.15)
UVR monitor	1.52 (0.15)	1.48 (0.13)	1.73 (0.15)
SPH Index (above items combined)			
Control	2.42 (0.08)	2.35 (0.07)	2.48 (0.07)
SunSmart App	2.47 (0.07)	2.41 (0.08)	2.61 (0.07)
UVR monitor	2.36 (0.08)	2.42 (0.09)	2.49 (0.08)

Scale: 1=never/rarely 2=sometimes 3=often 4=always
[a]mean= Estimated Marginal Means from GEE model

Table S2 Proportion of participants who improved their use of sunscreen

Timepoint	Yes[a] % (n)	No % (n)	OR	(95% CI)	p-value
**During Intervention[b]**					
**Sunscreen Use over weekday and weekend**					
Control	19.4 (7)	80.6 (29)	1.00		ref
SunSmart App	30.6 (11)	69.4 (25)	1.491	(0.482, 4.608)	0.488
UVR monitor	13.2 (5)	86.8 (33)	0.637	(0.181, 2.238)	0.482
**Sunscreen Use Weekday**					
Control	19.4 (7)	80.6 (29)	1.00		ref
SunSmart App	33.3 (12)	66.7 (24)	1.900	(0.635, 5.684)	0.251
UVR monitor	26.3 (10)	73.7 (28)	1.480	(0.494, 4.434)	0.484
**Sunscreen Use Weekend**					
Control	27.8 (10)	72.2 (26)	1.00		ref
SunSmart App	36.1 (13)	63.9 (23)	1.206	(0.427, 3.408)	0.724
UVR monitor	18.4 (7)	81.6 (31)	0.593	(0.190, 1.850)	0.368
**1 week after the intervention[c]**					
**Sunscreen Use over weekday and weekend**					
Control	21.6 (8)	78.4 (29)	1.00		ref
SunSmart App	33.3 (12)	66.7 (24)	1.508	(0.513, 4.435)	0.456
UVR monitor	13.5 (5)	86.5 (32)	0.562	(0.164, 1.930)	0.360
**Sunscreen Use Weekday**					
Control	13.5 (5)	86.5 (32)	1.00		ref
SunSmart App	33.3 (12)	66.7 (24)	2.808	(0.854, 9.238)	0.089
UVR monitor	10.8 (4)	89.2 (33)	0.776	(0.191, 3.152)	0.723
**Sunscreen Use Weekend**					
Control	24.3 (9)	75.7 (28)	1.00		ref
SunSmart App	30.6 (11)	69.4 (25)	1.278	(0.445, 3.673)	0.649
UVR monitor	10.8 (4)	89.2 (33)	0.377	(0.105, 1.357)	0.135
**12 weeks after the intervention[d]**					
**Sunscreen Use over weekday and weekend**					
Control	33.3 (12)	66.7 (24)	1.00		ref
SunSmart App	37.1 (13)	62.9 (22)	0.879	(0.310, 2.491)	0.808
UVR monitor	27.8 (10)	72.2 (26)	0.756	(0.268, 2.137)	0.598
**Sunscreen Use Weekday**					
Control	30.6 (11)	69.4 (25)	1.00		ref
SunSmart App	34.3 (12)	65.7 (23)	0.839	(0.286, 2.457)	0.749
UVR monitor	16.7 (6)	83.3 (30)	0.407	(0.122, 1.357)	0.144
**Sunscreen Use Weekend**					
Control	33.3 (12)	66.7 (24)	1.00		ref
SunSmart App	31.4 (11)	68.6 (24)	0.790	(0.282, 2.211)	0.653
UVR monitor	33.3 (12)	66.7 (24)	1.000	(0.370, 2.700)	0.999

[a] Yes = increase in percentage of days sunscreen used compared to baseline
[b] n=110 for the intervention measurement period
[c] n=109 for the 1 week after intervention measurement period
[d] n=107 for the 12 weeks after intervention measurement period

Table S3 Proportion of participants who increased their physical activity levels

Timepoint	Yes[a] % (n)	No % (n)	OR	(95% CI)	p-value
**During Intervention[b]**					
Control	36.1 (13)	63.9 (23)	1.00		ref
SunSmart App	30.6 (11)	69.4 (25)	0.771	(0.283, 2.098)	0.611
**1 week after the intervention[c]**					
Control	29.7 (11)	70.3 (26)	1.00		ref
SunSmart App	16.7 (6)	83.3 (30)	0.442	(0.140, 1.398)	0.165
**12 weeks after the intervention[d]**					
Control	41.7 (15)	58.3 (21)	1.00		ref
SunSmart App	28.6 (10)	71.4 (25)	0.555	(0.202, 1.527)	0.255
**During Intervention[b]**					
Control	36.1 (13)	63.9 (23)	1.00		ref
UVR monitor	23.7 (9)	76.3 (29)	0.547	(0.199, 1.505)	0.243
**1 week after the intervention[c]**					
Control	29.7 (11)	70.3 (26)	1.00		ref
UVR monitor	37.8 (14)	62.2 (23)	1.439	(0.546, 3.791)	0.462
**12 weeks after the intervention[d]**					
Control	41.7 (15)	58.3 (21)	1.00		ref
UVR monitor	38.9 (14)	61.1 (22)	0.891	(0.347, 2.287)	0.810

[a] Yes = increased percentage of days any physical activity undertaken compared to baseline
[b] n=110 for the intervention measurement period
[c] n=109 for the 1 week after intervention measurement period
[d] n=107 for the 12 weeks after intervention measurement period

Table S4 Proportion of participants reporting high risk unprotected torso sun exposure

	Yes[a] % (n)	No[b] % (n)	OR (95% CI)[3]	p-value
**Baseline**				
Control	23.1 (9)	76.9 (30)	1.00	ref
SunSmart App	22.5 (9)	77.5 (31)	1.167 (0.398, 3.421)	0.779
**End of Intervention**				
Control	36.1 (13)	63.9 (23)	1.00	ref
SunSmart App	22.2 (8)	77.8 (28)	1.939 (0.532, 7.064)	0.316
**1 week Follow-up**				
Control	24.3 (9)	75.7 (28)	1.00	ref
SunSmart App	19.4 (7)	80.6 (29)	1.483 (0.472, 4.659)	0.500
**3 month Follow-up**				
Control	27.8 (10)	72.2 (26)	1.00	ref
SunSmart App	31.4 (11)	68.6 (24)	1.070 (0.367, 3.118)	0.901
**Baseline**				
Control	23.1 (9)	76.9 (30)	1.00	ref
UVR monitor	23.1 (9)	76.9 (30)	1.000 (0.349, 2.869)	0.999
**End of Intervention**				
Control	36.1 (13)	63.9 (23)	1.00	ref
UVR monitor	21.1 (8)	78.9 (30)	2.102 (0.744, 5.935)	0.161
**1 week Follow-Up**				
Control	24.3 (9)	75.7 (28)	1.00	ref
UVR monitor	13.5 (5)	86.5 (32)	2.064 (0.617, 6.906)	0.240
**3 months Follow-Up**				
Control	27.8 (10)	72.2 (26)	1.00	ref
UVR monitor	16.7 (6)	83.3 (30)	2.019 (0.619, 6.590)	0.244

[a]Yes= average daily minutes of unprotected torso exposure for the measurement period was less than baseline

[b] No= average daily minutes of unprotected torso exposure for the measurement period was higher than baseline or did not change 5% below baseline score

n=118 participants with sun diary data

Table S5 Proportion of participants who recorded a sunburn

	Yes[d] % (n)	No[e] % (n)	OR (95% CI)[c]	p-value
**Baseline[a]**				
Control	12.8 (5)	87.2 (34)	1.00	ref
SunSmart App	22.5 (9)	77.5 (31)	0.491 (0.145, 1.662)	0.253
**End of Intervention[b]**				
Control	30.6 (11)	69.4 (25)	1.00	ref
SunSmart App	38.9 (14)	61.1 (22)	0.691 (0.256, 1.869)	0.467
**1 week Follow-Up[a]**				
Control	16.2 (6)	83.8 (31)	1.00	ref
SunSmart App	22.2 (8)	77.8 (28)	0.643 (0.192, 2.153)	0.474
**3 month Follow-up[a]**				
Control	16.7 (6)	83.3 (30)	1.00	ref
SunSmart App	28.6 (10)	71.4 (25)	0.495 (0.153, 1.597)	0.239
**Baseline[a]**				
Control	12.8 (5)	87.2 (34)	1.00	ref
UVR monitor	23.1 (9)	76.9 (30)	0.488 (0.147, 1.624)	0.242
**End of Intervention[b]**				
Control	30.6 (11)	69.4 (25)	1.00	ref
UVR monitor	42.1 (16)	57.9 (22)	0.602 (0.230, 1.570)	0.299
**1 week Follow-Up[a]**				
Control	16.2 (6)	83.8 (31)	1.00	ref
UVR monitor	16.2 (6)	83.3 (31)	1.000 (0.290, 3.450)	0.999
**3 months Follow-Up[a]**				
Control	16.7 (6)	83.3 (30)	1.00	ref
UVR monitor	19.4 (7)	80.6 (29)	0.824 (0.243, 2.797)	0.756

[a]= Measurement period 2 week duration, [b]= Measurement period 4 week duration.
[c]odds of 1 or more sunburns in the intervention groups compared to the control group.
[d]Yes= 1 or more sunburns recorded during measurement period.
[e] No= sunburn not recorded during measurement period.
n=118 participants with sun diary data.

Table S6 Proportion of participants who recorded intentionally seeking a suntan

	Suntan			
	Yes[d] % (n)	No[e] % (n)	OR (95% CI)[c]	p-value
**Baseline[a]**				
Control	7.7 (3)	92.3 (36)		ref
SunSmart App	10.0 (4)	90.0 (36)	0.955 (0.193, 4.717)	0.954
**Intervention [b]**				
Control	19.4 (7)	80.6 (29)		ref
SunSmart App	8.3 (3)	91.7 (33)	2.901 (0.669, 12.577)	0.155
**1 week Follow-Up[a]**				
Control	10.8 (4)	89.2 (33)		ref
SunSmart App	8.3 (3)	91.7 (33)	1.534 (0.309, 7.622)	0.601
**3 month Follow-up[a]**				
Control	8.3 (3)	91.7 (33)		ref
SunSmart App	17.1 (6)	82.9 (29)	0.575 (0.127, 2.603)	0.473
**Baseline[a]**				
Control	7.7 (3)	92.3 (36)		ref
UVR monitor	7.7 (3)	92.3 (36)	1.000 (0.182, 5.507)	0.999
**Intervention[b]**				
Control	19.4 (7)	80.6 (29)		ref
UVR monitor	21.1 (8)	78.9 (30)	0.876 (0.277, 2.770)	0.822
**1 week Follow-Up[a]**				
Control	10.8 (4)	89.2 (33)		ref
UVR monitor	5.4 (2)	94.6 (35)	2.142 (0.364, 12.623)	0.400
**3 months Follow-Up[a]**				
Control	8.3 (3)	91.7 (33)		ref
UVR monitor	5.6 (2)	94.4 (34)	1.575 (0.238, 10.437)	0.638

[a]= Measurement period 2 week duration, [b]= Measurement period 4 week duration.
[c]odds of 1 or more suntans in the intervention groups compared to the control group.
[d]Yes= seeking a suntan 1 or more times during measurement period.
[e] No= seeking a suntan not recorded during measurement period.
n=118 participants with sun diary data.

	SunSmart App n=35		UVR Monitor n=36	
	%	(n)	%	(n)
Did you find the device helpful to guide your sun protective behaviour?				
Yes	65.7	(23)	63.9	(23)
No	34.3	(12)	36.1	(13)
Did you change or modify your behaviour in response to the output from the device?				
Yes	54.3	(19)	47.2	(17)
No	45.7	(16)	52.8	(19)
Would you purchase or download the device after the study?				
Yes	40.0	(14)	19.4	(7)
No	60.0	(21)	80.6	(29)
Would you recommend the device to a friend or family member?				
Yes	65.7	(23)	52.8	(19)
No	34.3	(12)	47.2	(17)
The device was encouraging				
Agree/Strongly Agree	62.9	(22)	47.2	(17)
Unsure	17.1	(6)	16.7	(6)
Disagree/Strongly Disagree	20.0	(7)	36.1	(13)
The device was repeating what I already know				
Agree/Strongly Agree	60.0	(21)	36.1	(13)
Unsure	17.1	(6)	25.0	(9)
Disagree/Strongly Disagree	22.9	(8)	36.1	(13)
Missing	-	-	2.8	(1)

Table S7 Satisfaction with Intervention Devices
